# Supplementary material for: Integrated Amino Acids and Transcriptome Analysis Reveals Arginine Transporter SLC7A2 Is a Novel Regulator of Myogenic Differentiation
Source: Int J Mol Sci. 2023 Dec 20;25(1):95. doi: 10.3390/ijms25010095 (PMC10778648; doi:10.3390/ijms25010095)
Supplement: Supplementary file 1 [file ijms-25-00095-s001.zip › Supplementary Figure S1.pdf]

Supplementary Materials

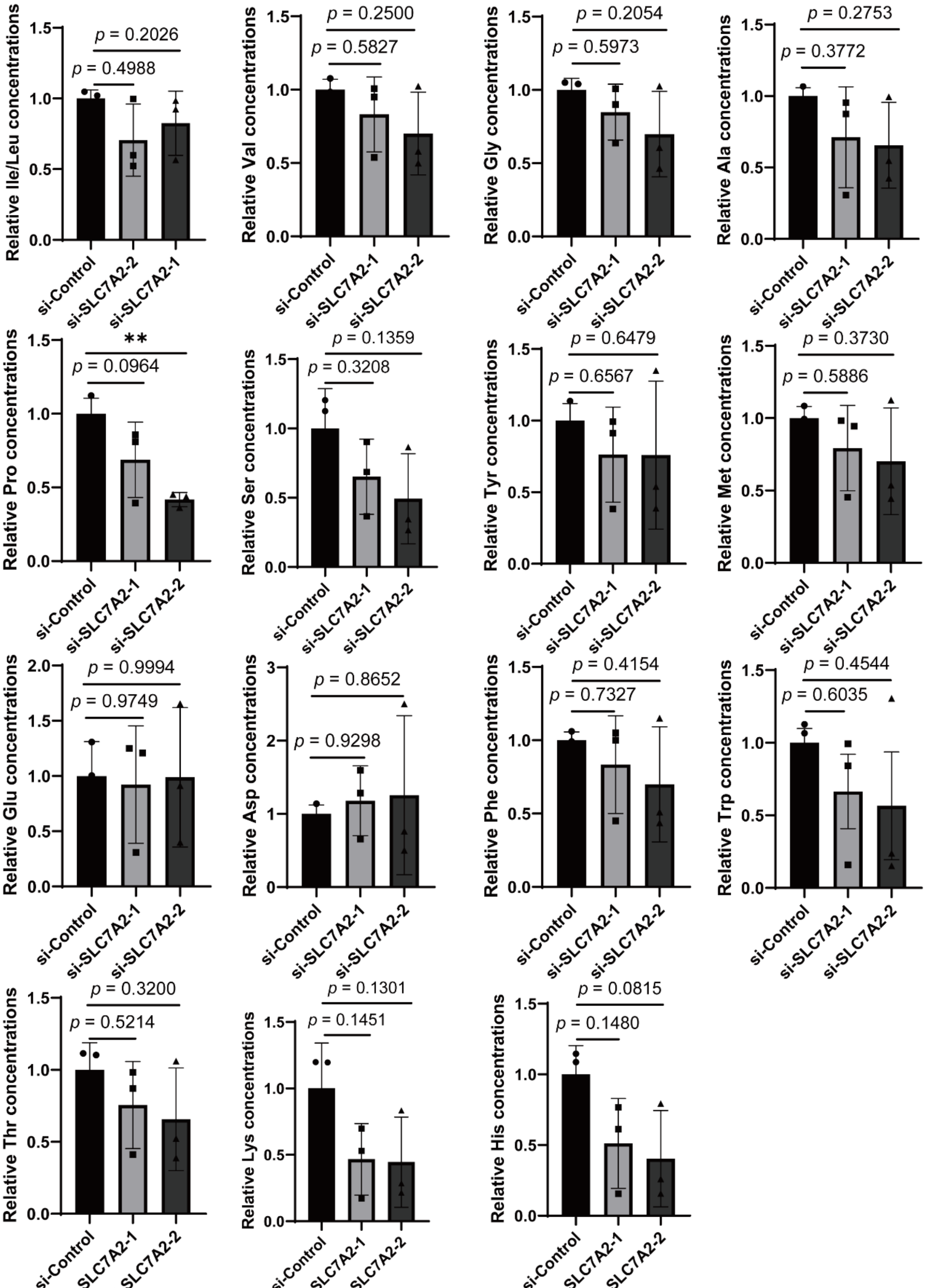

Figure S1. Amino acid changes after SLC7A2 knockdown in C2C12 cells (n = 3). The bars represent the mean ± SD. **, $p < 0.01$. Statistical significance determined using one-way ANOVA.
